# Fish tank granuloma: An emerging skin disease in Iran mimicking Cutaneous Leishmaniasis

**DOI:** 10.1371/journal.pone.0221367

**Published:** 2019-09-19

**Authors:** Abdolmajid Fata, Amin Bojdy, Masoud Maleki, Bibi Razieh Hosseini Farash, Kiarash Ghazvini, Parastoo Tajzadeh, Vida Vakili, Elham Moghaddas, Pietro Mastroeni, Shadi Rahmani

**Affiliations:** 1 Cutaneous leishmaniasis Research Center, Mashhad University of Medical Sciences, Mashhad, Khorasan-e-Razavi, Iran; 2 Department of Parasitology and Mycology, Faculty of Medicine, Mashhad University of Medical Sciences, Mashhad, Khorasan-e-Razavi, Iran; 3 Department of Infectious Diseases, Imam Reza Hospital, Mashhad University of Medical Sciences, Mashhad, Khorasan-e-Razavi, Iran; 4 Department of Dermatology, Imam Reza Hospital, Mashhad University of Medical Sciences, Mashhad, Khorasan-e-Razavi, Iran; 5 Department of Microbiology, Quem Hospital, Mashhad University of Medical Sciences, Mashhad, Khorasan-e-Razavi, Iran; 6 Department of Medical Lab Sciences, Faculty of nursing, Kashmar, Mashhad University of Medical Sciences, Mashhad, Khorasan-e-Razavi, Iran; 7 Department of Social Medicine, Faculty of Medicine, Mashhad University of Medical Sciences, Mashhad, Khorasan-e-Razavi, Iran; 8 Department of Veterinary Medicine, University of Cambridge, Cambridge, United Kingdom; Universita degli Studi di Parma, ITALY

## Abstract

**Objective:**

*Mycobacterium marinum* causes a rare cutaneous disease known as fish tank granuloma (FTG). The disease manifestations resemble those associated with Cutaneous Leishmaniasis (CL). The aim of this study was to determine whether FTG was the cause of cutaneous lesions in patients who were referred to the Parasitology laboratory of Imam Reza Hospital in Mashhad to be investigated for CL.

**Materials/Methods:**

One hundered patients, clinically diagnosed with CL between April 2014 and March 2015, were included in this study. Ziehl-Neelsen staining was performed to identify acid-fast *Mycobacterium* in addition to bacterial cultures using Löwenstein-Jensen medium. Skin lesion samples were also collected and kept on DNA banking cards for PCR testing.

**Results:**

Twenty-nine of the 100 individuals with skin lesions, and therefore suspected of suffering from CL, tested positive for *Mycobacterium marinum* by PCR. Of these, 21 (72.4%) were male and 8(27.6%) were female. In 97% of these cases the lesions were located on hands and fingers. These patients had a history of manipulating fish and had been in contact with aquarium water. A sporotrichoid appearance was observed in 58.6% of the patients with mycobacterial lesions; 67% of patients had multiple head appearance.

**Conclusion:**

Patients suspected to have CL and who test negative for CL could be affected by FTG. Therefore, after obtaining an accurate case history, molecular diagnosis is recommended for cases that give a negative result by conventional methods.

## Introduction

*Mycobacterium marinum* is a free-living organism and fish-associated pathogen organism, which is commonly found in fresh or salt water. This bacterium causes rare cutaneous infections in humans. The organism penetrates the skin and induces granulomas or sporotrichoid lymphocutaneous lesions, known as fish tank granuloma (FTG). The infection usually occurs in individuals who come in close contact with the contents of aquariums [1,2]. FTG can also be considered an occupational disease since it has recently been diagnosed in a sushi preparer [3]. Moreover, *Mycobacterium marinum* could threaten people who use fish pedicure methods [4]. The cutaneous form of the disease consists of single or multiple skin lesions. However a disseminated form has been reported in immune-compromised patients[5]. The skin lesions caused by *M*. *marinum* develop on the hands, fingers, feet and knees after an incubation period of 3–4 weeks. The lesions usually appear in those sites of the body that come in contact with contaminated water of swimming pools or aquaria[6,7].

A reasonable approach to the management of *M*. *marinum* consists of treatment with two active agents for one to two months after resolution of symptoms (total duration is typically three to four months)[8]. Therapy could include clarithromycin together with either ethambutol or rifampin. Mild disease can be managed with single-drug therapy using clarithromycin, minocycline, doxycycline or trimethoprim-sulfamethoxazole [9,10]. Despite there is no standard therapy of choice for FTG, a large cohort of patients have been successfully treated with clarithromycin monotherapy [3]. The clinical features of FTG are similar to those of cutaneous leishmaniasis (CL), especially in the sporotrichotic form. Thus, it is often arduous to differentiate between these two diseases by simple observation of clinical signs and FTG is often misdiagnosed as CL in endemic areas[11]

CL is one of the most important cutaneous parasitic skin diseases in Middle East, including Iran[12]. *Leishmania tropica* and *Leishmania major* are the two common agents of CL in Iran. The broad spectrum of clinical manifestations for CL ranges from a simple papules, erythematous nodules to less common papillomatous or verrucous lesions and to the sporotrichoid form [13]. The host immune response, the complexity of the organism, the environment, and the species of *Leishmania* are considered as the main determinants of the appearance of the skin lesions [14].

Clinical features and laboratory tests facilitate the diagnosis of CL. Numerous diagnostic techniques (direct microscopy, serology and molecular methods) are currently used and these vary in sensitivity and specificity. Parasitological examinations and observation of amastigotes by Giemsa-stained smears, biopsy and culture are the conventional and routine methods for the diagnosis of CL [15]. The detection of *M*. *marinum* in acid-fast stained smears, biopsies, bacteriological cultures and DNA-based methods may be helpful to identify FTG [16,17].

The purpose of this study was to identify the causative agents of disease by direct smears and molecular methods in samples obtained from patients who had chronic and atypical skin lesions clinically diagnosed as CL.

## Materials and methods

### Ethical considerations

This project was approved by the Ethical Committee of Mashhad University of Medical Sciences, Code No. IR, MUMS, REC, 1393.960, in accordance with the ethical principles of Helsinki Declaration. The skin lesion samples were obtained from adult patients who signed informed consent forms. In the case of children, the informed consent forms were signed by their parents or guardians.

### Study population and sampling

Between April 2014 and March 2015, more than 1500 patients suspected to be suffering from CL attended the Cutaneous Leishmaniasis Research Center in Mashhad. This is one of the main endemic areas for anthroponotic cutaneous leishmaniasis (ACL). During the last two decades, CL cases have significantly risen in Mashhad that is located in Khorasan-e-Razavi, one of the provinces in Iran with highest prevalence of CL[18].

One hundred individuals with chronic skin lesions, with or without lymphadenopathy and lymphangitis and with a history of aquatic exposure, were included in this study. Demographic and clinical information were recorded.

Three samples were obtained from the skin lesions of each patient to test for CL and FTG; the first sample was put on a DNA banking card (Kawsar DBC^™^, Iran); a second sample was used to prepare smears for direct examination by Giemsa and Ziehl-Neelsen staining. After direct microscopy, the smears were kept for DNA extraction to compare with DNA extracted from DBC^™^. A third sample was cultured in Löwenstein-Jensen medium at 30 to 32°C for 3–4 weeks.

### Molecular analysis

#### DNA extraction

DNA was extracted from the DNA banking cards and from direct smears following the protocols indicated in the DBC^™^ (Kawsar, Iran) and DNA extraction kit (Genet Bio, Korea), respectively. All the DNA samples were stored at -20°C until they were used in PCR assays.

#### Amplification of *Leishmania* DNA by conventional PCR

Partial sequences of *Leishmania* kinetoplast DNA minicircle were amplified using the following primers: forward, F (5’- TCGCAGAACGCCCCTACC -3’), reverse, R (5’- AGGGGTTGGTGTAAAATAGG- 3’) [19]. DNA amplification included an initial step at 95°C for 5 min followed by 38 cycles at 94°C for 30 s, 60°C for 45 s, 72°C for 60 s and a final extension step at 72°C for 7 min. The bands of interest for *L*. *tropica* and *L*. *major* were 744 bp and 615 bp respectively. The PCR mixture included 1.5 μl (5pm) of each primer, 0.5μl (5U/μl) Taq DNA polymerase, 0.5 μl dNTPs, 0.5 μl Mgcl2 and 1 μl DNA for each 25μl reaction.

#### Amplification of *Mycobacterium* DNA by nested PCR

The partial sequence of heat shock protein 65(*hsp 65*) was targeted to identify *Mycobacterium* spp by nested PCR according to Wu *et al*. [20]. Primers M1 (5′-CCCCACGATCACCAACGATG-3′) and M4 (5′-CGAGATGTAGCCCTTGTCGAACC-3′) formed a 463-bp product in the first round of amplification with 5 μl of DNA template in a 20 *μ*l reaction mixture containing 0.4 *μ*l of each primers, 0.2μl (2U/μl) Taq DNA polymerase, 0.5μl dNTPs, 1.5 μl Mgcl2, 2.5 μl 10X PCR and 14.5 μl ddH2O. 3 μl of the first amplification PCR product were used to perform nested PCR using TB11 (5′-ACCAACGATGGTGTGTCCAT-3′) and TB12 (5′-CTTGTCGAACCGCATACCCT-3′) in 25 μl of the reaction mixture yielding a band of 439 bp [21]. All the PCR assays included a positive and negative control and were carried out using a ASTEC thermal cycler (ASTEC-PC818, Japan). The purified PCR products were sequenced by a commercial company (Pishgam, Tehran, Iran) to determine the species of *Mycobacterium*. Then, the partial sequences of heat shock protein 65(*hsp 65*) were compared with similar sequences of this organisms in Gene Bank using the BLAST online software of the National Center for Biotechnology Information.

### Statistical analysis

Results were analyzed using the SPSS software (IBM SPSS Statistics 24) and the significant differences were determined by Fisher's exact test and independent t-test with 95% confidence interval.

## Results

### Direct smear examination

**Fig 1:**
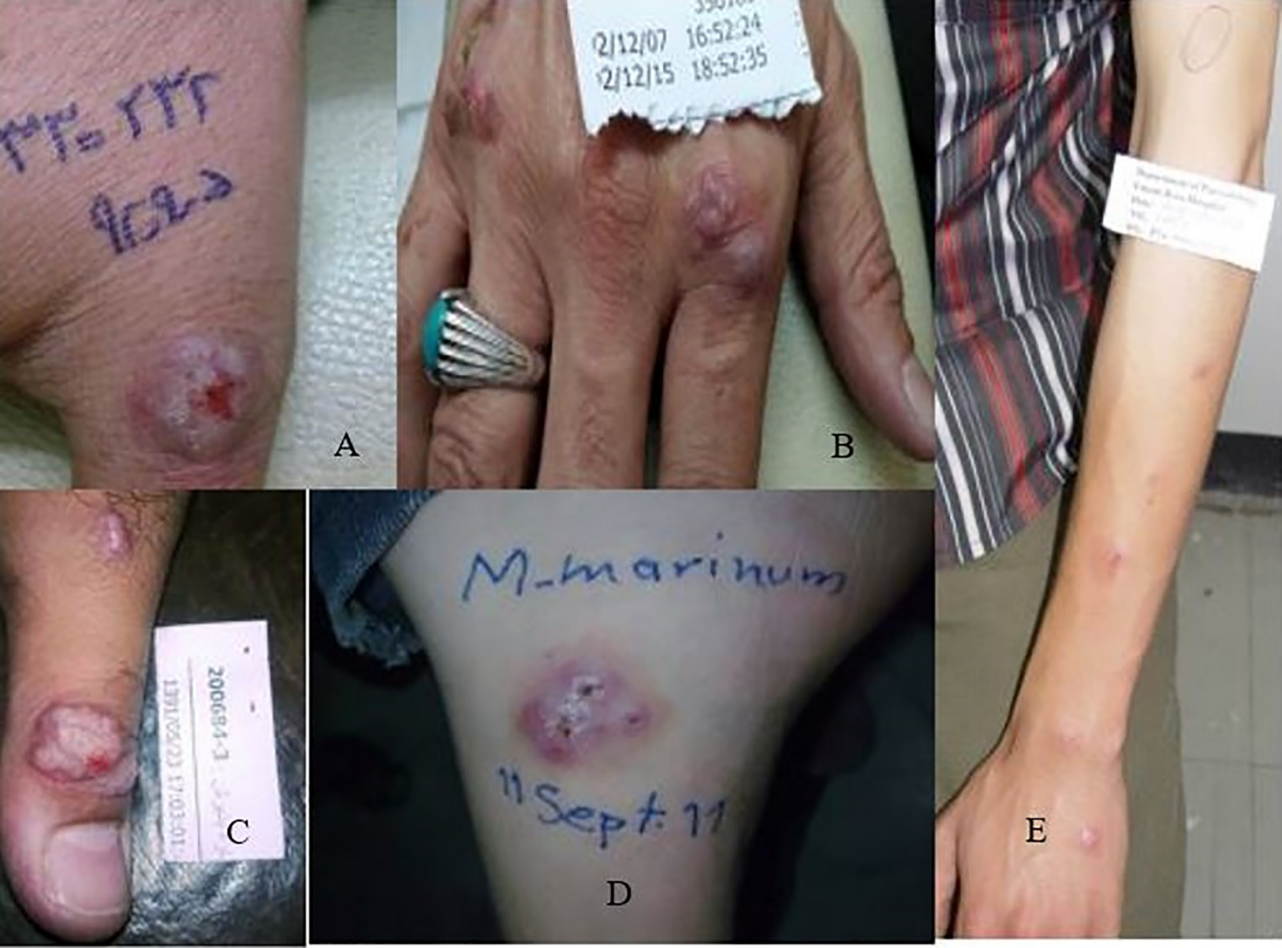
(A, B, C) *Mycobacterium marinum* Nodular lesions in the fingers and hands; (D) *Mycobacterium marinum* papular lesions on the foot;(E) sporotrichoid form of *Mycobacterium marinum* lesions on the arm of a patient.

**Fig 2:**
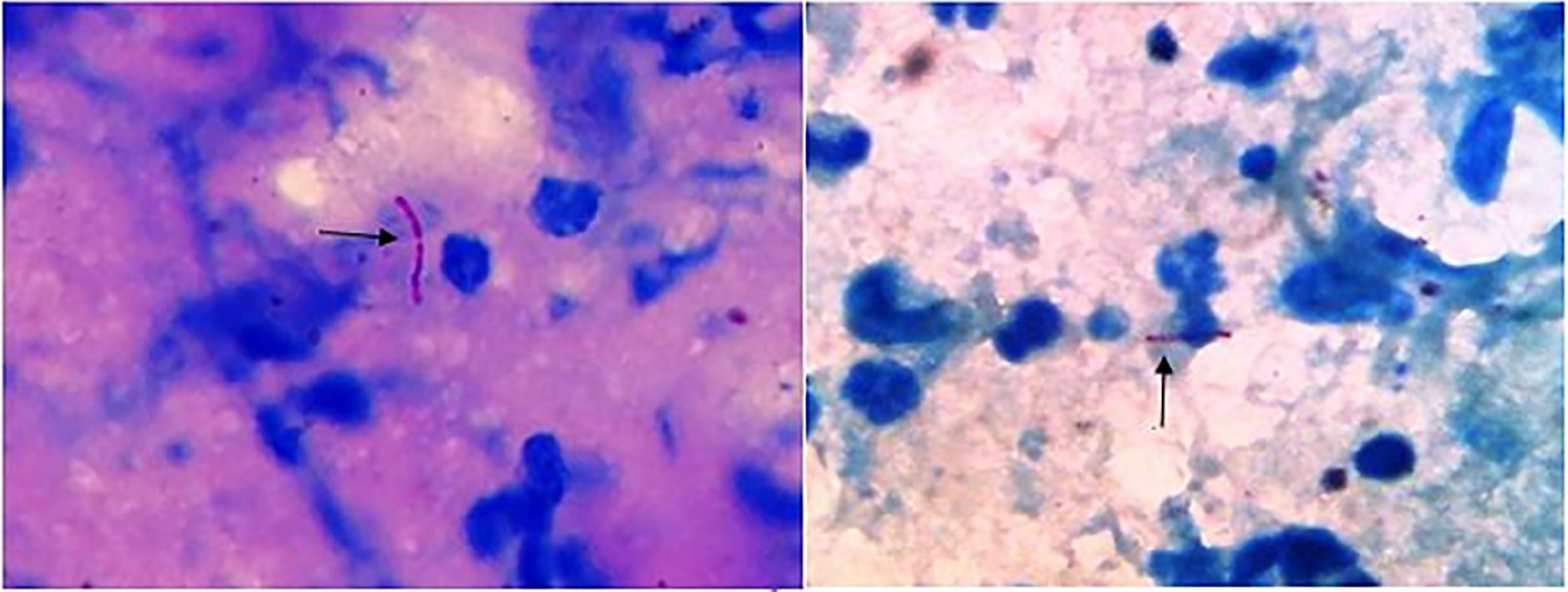
The presence of Acid-fast bacilli (Ziehl-Neelsen staining) in direct smears in lesion scrapping of patients infected by *Mycobacterium marinum*, ×100 objective.

A total of 100 patients with skin lesions suspected to be indicative of CL were included in the study. 63% of these patients were male and 37% were female with mean age of 31.3±3.4. The amastigote forms of *Leishmania* were observed in direct smears obtained from the lesions of 45 individuals (45%), while acid-fast bacilli were identified in 21 smears (21%) (Figs 1 and 2).

### Culture

**Fig 3:**
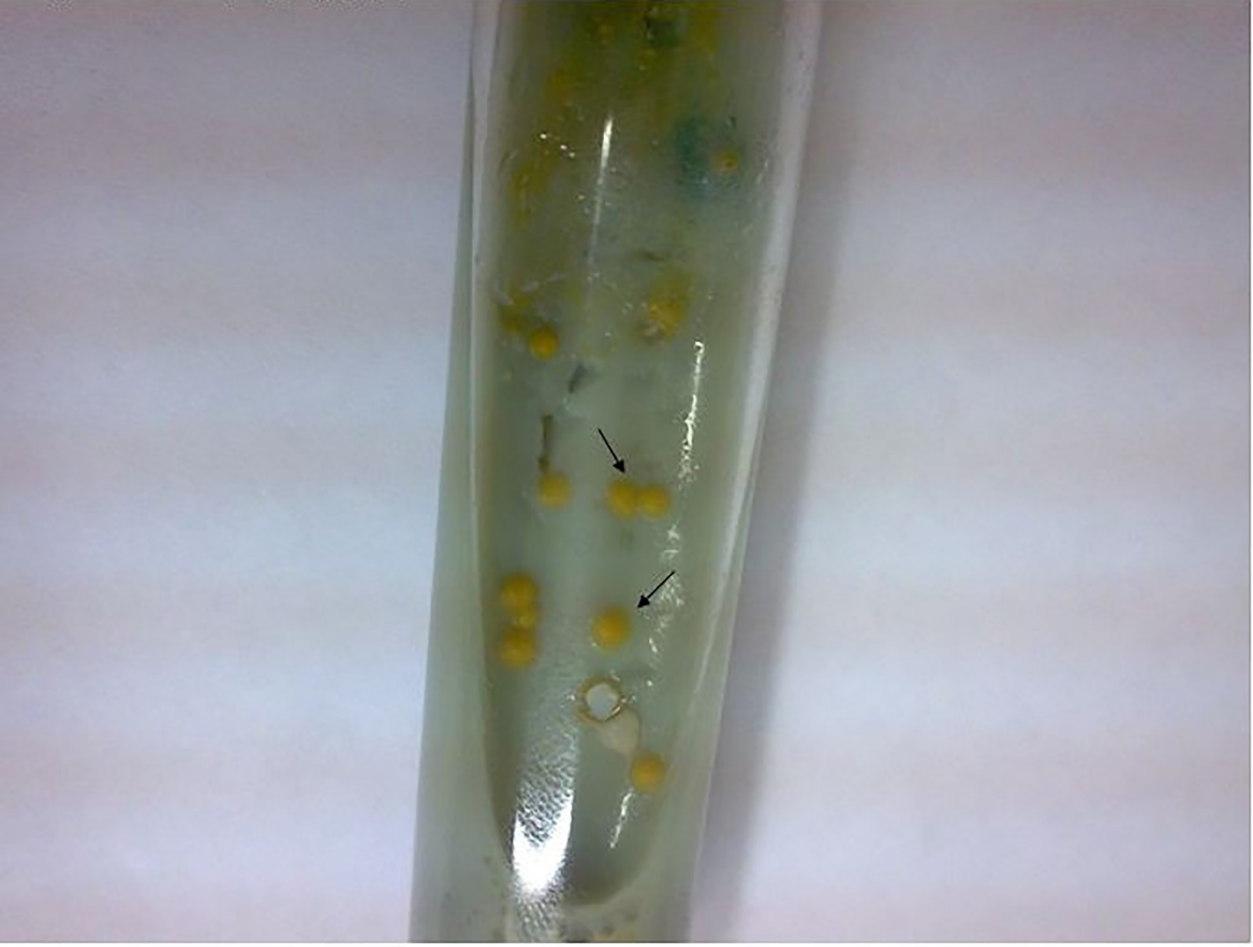
Photochromic colonies of *Mycobacterium marinum* in Löwenstein-Jensen medium, after approximately 14 days of incubation at 30°C.

Only 15 of the skin samples tested showed mycobacterial growth on Löwenstein-Jensen medium (Fig 3).

### Molecular analysis

**Fig 4:**
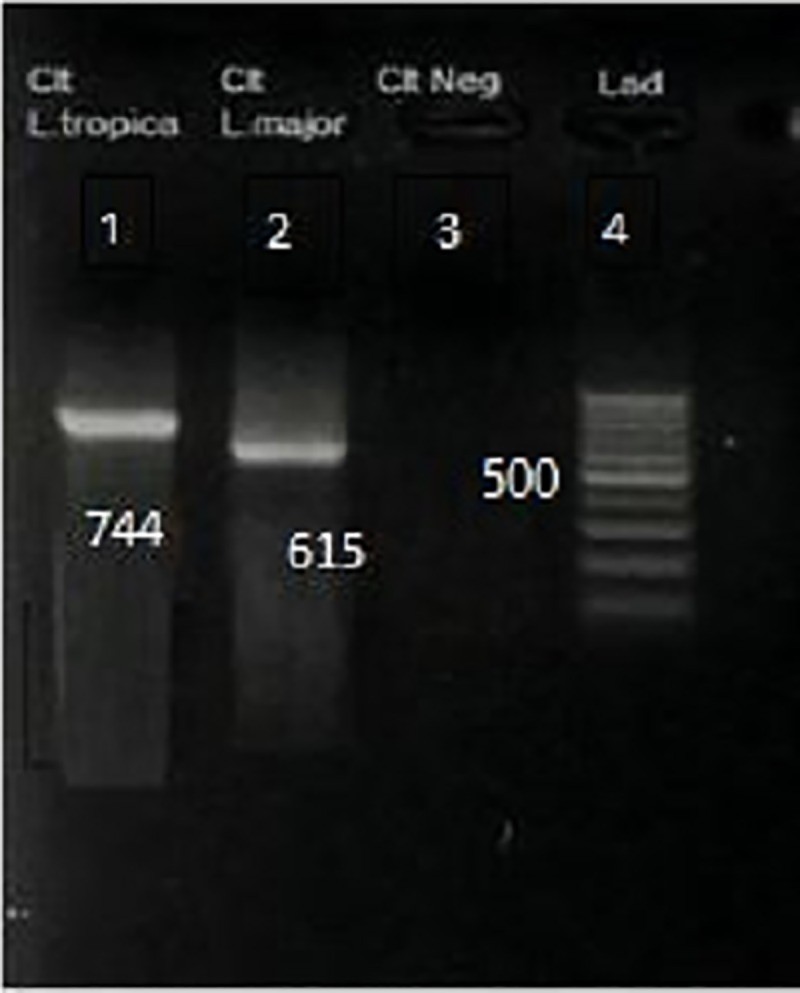
PCR results of amplification of leishmanial Kinetoplast DNA. Left to right: lane 1, *L*. *tropica*; lane 2, *L*. *major*; lane 3, negative control; lane 4, molecular-weight standards.

PCR amplification of DNA extracted from DNA banking cards and direct slides demonstrated gave a positive result for *Leishmania* in 49 and 31 cases, respectively. The PCR products showed a 744 bp band for *L*. *tropica* (45%) and 615 bp band for *L*. *major* (4%) in 26 males and 23 female patients (Fig 4).

**Fig 5:**
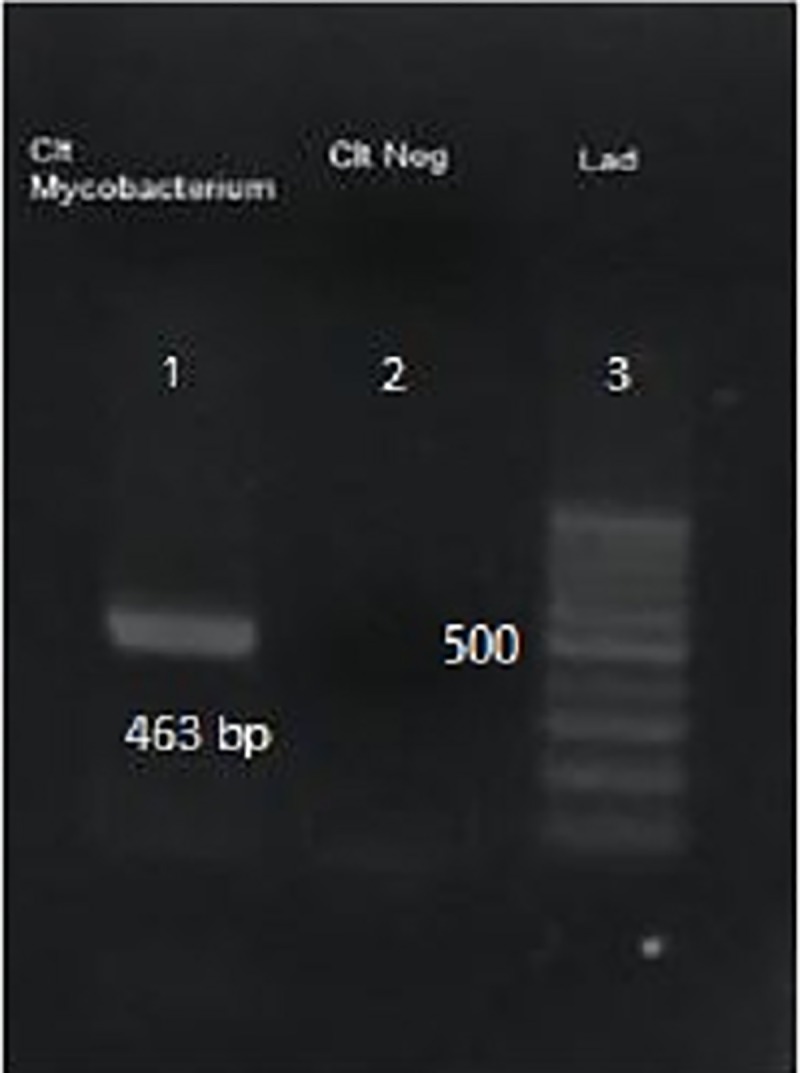
PCR results of the first amplification of partial sequence of mycobacterial hsp 65 with M1 & M4 primers. Left to right: lane 1, *Mycobacterium* spp.; lane 2, negative control; lane 3, molecular-weight standards.

**Fig 6:**
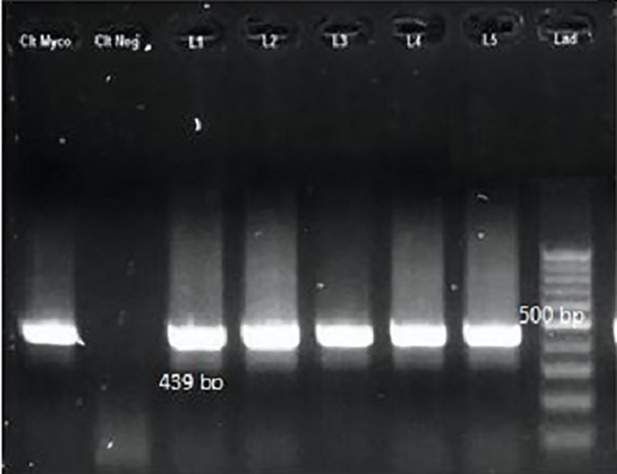
Nested-PCR results of *Mycobacterium* spp. with TB11 & TB12 primers. Left to right: positive control; negative control; lanes L1, L2, L3, L4 and L5 *M*. *marinum*; lane 6, molecular-weight standards.

Nested PCR on DNA extracted from the direct smears, 29 samples showed bands of interest corresponding to *Mycobacterium* DNA (Figs 5 and 6). Nevertheless, the nested PCR results were negative in all of the DNA samples obtained from DNA banking card. The sequences of the PCR products of the 29 positive samples, consisting of samples from 21 males and 8 females, showed 99% to 100% homology to published *M*. *marinum* sequences.

### Clinical and epidemiological features

All of the patients with FTG had a history of close contact with aquarium water and suffered from painful lesions. Sporotrichoid and multiple-head clinical forms were observed in 58.6% and 68.9% of the patients with mycobacterial lesions, respectively. In this study, the sporotrichotic form has only been reported in one CL case and 5 patients had painful CL ulcers.

All the FTG patients were treated with clarithromycin with or without rifampin [3]. The treatment was continued for 1–2 months after the resolution of lesions and was successful.

The clinical signs, laboratory tests/results and epidemiological features used for the diagnosis of FTG and CL are shown in Table 1.

**Table 1:** The clinical, laboratory test and epidemiological features of patients with FTG and CL.

		CL	p-value(CL cases)	FTG	p-value(FTG cases)	p-value(CL &FTG)
**Gender**	Male	26 (53%)	P > 0.05	21(73.4%)	P > 0.05	
	Female	23 (47%)		8 (27.6%)		
	Missing data	0		0		
**Age (years)**	1–10 years	3 (6.1%)	P > 0.05	1 (3.4%)	P > 0.05	
	10–20 years	6 (12.2%)		3 (10.3%)		
	20–30 years	14 (28.6%)		7 (24.1%)		
	30–40 years	20 (40.8%)		14 (48.4%)		
	40–50 years	5 (10.2%)		2 (6.9%)		
	>50 years	1 (2%)		2 (6.9%)		
**Occupation**	Student	9 (18.4%)	P > 0.05	2 (6.9%)	P > 0.05	
	Employee	12 (24.5%)		6 (20.7%)		
	Self-employed	9 (18.4%)		4 (13.8%)		
	Stockman	4 (8.1%)		0		
	House-wife	9 (18.4%)		4(13.8%)		
	Aquarist	0		9 (31%)		
	others	6 (12.2%)		4(13.8%)		
**Affected site of body**	Hands	21 (42.8%)	P > 0.05	28 (96.5%)	P < 0.05	
	Feet	9 (18.4%)		1 (3.5%)		
	Face	12 (24.5%)		0		
	Others	7 (14.3%)		0		
**The site of the lesions on the hand**	Fingers and hands	12(57.2%)	P > 0.05	23 (79.2%)	P < 0.05	
	Wrists	1 (4.8%)		2 (7.14%)		
	arms	8 (38%)		3 (10.35%)		
**The Morphology of lesions**	Ulcer	4 (8.2%)	P > 0.05	2 (6.9%)	P < 0.05	
	Nodule	13 (26.5%)		23 (79.3%)		
	Papule	32 (65.3%)		4 (13.8%)		
**Sporotrichoid form**	Yes	1 (2%)	P > 0.05	17 (58.6%)	P < 0.05	P < 0.05
	No	48 (98%)		12 (41.4%)		
**A history of contact with fish and aquarium water**	Yes	2 (4%)	P > 0.05	29 (100%)	P < 0.05	P < 0.05
	No	47 (96%)		0		
**Complaining of pain**	Yes	5 (10.2%)	P > 0.05	29 (100%)	P < 0.05	P < 0.05
	No	44 (89.8%)		0		
**Direct slide examination**	Positive	45 (45%)		21 (21%)		P > 0.05
	Not seen	51 (51%)		71 (71%)		
	Missing data	4 (4%)		8 (8%)		
**Culture**	Positive	No culture		15 (15%)		
	No growth			85 (85%)		
	Missing data			14 (14%)		
**Amplification results of DNA extraction using DNA banking card**	Positive	31 (31%)		0		P < 0.05
	Negative	18 (18%)		100 (100%)		
	Missing data			0		
**Amplification results of DNA extraction using direct slides**	Positive	49 (49%)		29 (29%)		
	Negative	51 (51%)		71 (71%)		
	Missing data	0		0		

## Discussion

Mashhad is a city located in the center of Khorasan-e-Razavi Province, in the North-Eastern part of Iran. Mashhad is an endemic area for CL[22]. Both anthroponotic and zoonotic forms of CL are present in this city, but with different frequencies[23]. Most of the patients suspected to suffer from CL are referred to the clinical parasitology laboratory for a conclusive diagnosis. Some of these patients have a history of previous direct examination with negative result. *M*. *marinum* is an atypical *Mycobacterium* that causes FTG in humans after trauma and exposure to aquatic environments. Using clinical criteria alone, it is difficult to differentiate between FTG and CL, sporotrichosis, tularemia, sarcoidosis, and deep fungal infections[24].

In the present study the presence of *M*. *marinum* was investigated in patients that were referred to the Cutaneous Leishmaniasis Research Center in Mashhad University of Medical Sciences because of cutaneous lesions indicative of CL.

Among 100 individuals with cutaneous lesions, *M*. *marinum* infection was observed in 29% of cases of which 28% were females and 72% males; CL was diagnosed in 49% of patients, of which 47% were females and 53% males, with the highest frequency of both diseases in the age group between 30 and 40 years old. There was no statistically significant correlation between gender, occupation and age in susceptibility to FTG and CL (Fisher's exact and independent t-tests (P > 0.05)). Our results are consistent with data reported in other studies [2,25–27].

All the patients with FTG suffered from painful ulcers (P < 0.05)[5]. This indicates that painful lesions are a clinical correlate of FTG and this may be considered as a parameter to differentiate between CL and FTG.

A history of close contact with fish tanks was reported by FTG patients. This indicates that the cutaneous form of *M*. *marinum* is associated with skin erosion during handling fish and/or cleaning fish tanks (P < 0.05).

The majority of FTG lesions had nodular presentation with an important statistical correlation between the sporotrichotic form and FTG (P < 0.05). Previous studies reported the sporotrichotic form of skin lesions in 20–40% of FTG patients. In the present study we found that the sporotrichoid form occurred in a higher percentage of FTG patients, being observed in approximately 60% of the patients [28–30].

According to previous studies, most of the lesions caused by *M*. *marinum* are localized in the upper extremities and in exposed parts of the body. For example FTG lesions have been frequently observed on the hands and fingers of aquarium owners [25]. The present study showed similar results with noticeable lesions located on the hands and fingers (P < 0.05) of the majority of patients. Conversely, in cutaneous leishmaniasis hands and face are usually the most affected body sites[31,32].

*M*. *marinum* was observed in direct smears from 72% patients. Despite histology and bacteriology techniques are common methods to identify FTG, nonspecific histological features during the early phases of the infection and improper sampling may lead to misdiagnosis [33,34]. Tissue culture obtained from biopsy samples is considered to be the gold standard method for the diagnosis of mycobacterial infections. The positivity rate of cultures has been reported to be between 70 and 80% for biopsy specimens of skin lesions[35,36]. In our study *M*. *marinum* was culturable from lesions of 50% of the FTG patients. Therefore, cultures from scraped samples and exudates of lesions could yield fewer positive results compared to tissue specimens.

However, the high specificity (100%) and acceptable sensitivity of about 50–70% of parasitological methods make this approach the first diagnostic option for the diagnosis of CL in endemic areas [37–39].

The high sensitivity (92%) of Giemsa-stained direct smears shown in this study, compared to previously reported figures, indicates the importance of sampling techniques and expert microscopic observations in the accurate diagnosis of CL.

Molecular techniques are considered the most sensitive methods to diagnose mycobacterial and leishmanial infections using lesion exudates and scraped samples. Due to good specificity in the detection of leishmanial DNA, PCR has become the reference test for diagnosis of leishmaniasis. On the contrary, false positives occur more often in the molecular diagnosis of *M*. *marinum* isolated from aquarium fish[37,40]. In our study the highest number of specific positive results (highest sensitivity) were observed when PCR amplification was performed on DNA extracted from direct slides for both FTG and CL. These results show concordance with data published by other investigators[41,42].

DNA extraction from whatman filter paper cards and DNA banking cards have been suggested as non-invasive and easy-to-perform methods for the molecular diagnosis of *Leishmania* infections[43]. In present study, two different methods were compared for *Leishmania* and *Mycobacteria* DNA extraction, using direct smears and DNA banking cards. Differently from previous data, only 61% of CL patients in this study tested positive after amplification from DNA extracted using DBC^™^ while all the samples of FTG were negative in the nested-PCR[44]. These findings indicated that DBC^™^ was not able to extract the mycobacterial DNA from the specimens. It appears that sufficient template may not be present in punches, but it would be present in direct smears. Regarding the presence of lipid complexes in the cell wall of *Mycobacterium*, it can also be speculated that the buffers used in DBC^™^ may not be able to extract adequate mycobacterial DNA. Moreover, DBC^™^ card had a lower sensitivity (61%) compared to direct smear DNA extraction in CL cases [45,46].

## Conclusion

The present study shows that FTG is a common disease that affects aquarium owners in Mashhad. Consequently, obtaining an accurate case history from the patients suspected to suffer from CL and that have with negative test result could often reveal FTG. Since the treatment of FTG is difficult and completely different from CL, treatment of skin lesion should not be based only on clinical signs. Therefore, molecular diagnosis is recommended for those patients where conventional methods yield negative results.

## Supporting information

S1 Table This is the SPSS file that supports the information of Table 1. (SAV)
